# A Phase I, open-label, randomized, crossover study in three parallel groups to evaluate the effect of Rifampicin, Ketoconazole, and Omeprazole on the pharmacokinetics of THC/CBD oromucosal spray in healthy volunteers

**DOI:** 10.1186/2193-1801-2-236

**Published:** 2013-05-24

**Authors:** Colin Stott, Linda White, Stephen Wright, Darren Wilbraham, Geoffrey Guy

**Affiliations:** GW Pharma Ltd, Porton Down Science Park Salisbury, Wiltshire, SP4 0JQ UK; Quintiles Drug Research Unit at Guy’s Hospital, 6 Newcomen Street, London, UK

**Keywords:** Cannabidiol, Cytochrome P450, Delta-9-tetrahydrocannabinol, Sativex ^®^^†^, Nabiximols^†^, THC/CBD spray

## Abstract

**Abstract:**

This Phase I study aimed to assess the potential drug-drug interactions (pharmacokinetic [PK] and safety profile) of Δ9-tetrahydrocannabinol (THC)/cannabidiol (CBD) oromucosal spray (Sativex ^®^, nabiximols) in combination with cytochrome P450 (CYP450) inducer (rifampicin) or inhibitors (ketoconazole or omeprazole).

Thirty-six healthy male subjects were divided into three groups of 12, and then randomized to one of two treatment sequences per group. Subjects received four sprays of THC/CBD (10.8/10 mg) alongside single doses of the CYP3A and 2C19 inducer rifampicin (600 mg), CYP3A inhibitor ketoconazole (400 mg) or CYP2C19 inhibitor omeprazole (40 mg). Plasma samples were analyzed for CBD, THC and its metabolite 11-hydroxy-THC (11-OH-THC).

A single dose of four sprays of THC/CBD spray (10.8/10 mg) following repeated doses of rifampicin (600 mg) reduced the C_max_ and AUC of all analytes. C_max_ reduced from 2.94 to 1.88 ng/mL (-36%), 1.03 to 0.50 ng/mL (-52%) and 3.38 to 0.45 ng/mL (-87%) for THC, CBD and 11-OH-THC, respectively compared to single dose administration of THC/CBD spray alone. Ketoconazole co-administration with THC/CBD spray had the opposite effect, increasing the C_max_ of the respective analytes from 2.65 to 3.36 ng/mL (+27%), 0.66 to 1.25 ng/mL (+89%) and 3.59 to 10.92 ng/mL (+204%). No significant deviations in C_max_ or AUC for any analyte were observed when THC/CBD spray was co-administered with omeprazole. THC/CBD spray was well tolerated by the study subjects both alone and in combination with rifampicin, ketoconazole and omeprazole.

Evaluation of the PKs of THC/CBD spray alone and in combination with CYP450 inhibitors/inducers suggests that all analytes are substrates for the isoenzyme CYP3A4, but not CYP2C19. On the basis of our findings, there is likely to be little impact on other drugs metabolized by CYP enzymes on the PK parameters of THC/CBD spray, but potential effects should be taken into consideration when co-administering THC/CBD spray with compounds which share the CYP3A4 pathway such as rifampicin or ketoconazole.

**Trials registration:**

NCT01323465

## Introduction

The endocannabinoid system modulator Δ9-tetrahydrocannabinol (THC)/cannabidiol (CBD) oromucosal spray (Sativex ^®^, nabiximols) has been reported to be effective in relieving a number of multiple sclerosis (MS) symptoms including spasticity, central neuropathic pain and bladder dysfunction (Rog et al. 2005; Johnson et al. 2010; Rog et al. 2007), and has recently been approved in various European countries and abroad (i.e. in Canada, Israel, New Zealand) as add-on treatment for spasticity in MS patients. Other potential indications for this compound include pain relief in advanced cancer (Johnson et al. 2010; Porteney et al. 2012), as well as peripheral neuropathic pain in MS (Nurmikko et al. 2007). Derived from proprietary cannabis plant varieties bred to exhibit a pre-determined content of cannabinoids (CBs), THC/CBD spray is fully standardized and contains two principal CBs, THC and CBD at an approximately 1:1 ratio as well as minor amounts of other CBs and non-CB components. The specified CBs constitute at least 90% of the total CB content of the extract, however, the minor CBs and other constituents also contribute to the therapeutic profile of THC/CBD spray (Russo 2011), and may be involved in stabilizing the extract (Whittle et al. 2001).

CBs are thought to act primarily via activation of specific CB receptors, CB_1_ and CB_2_ (Howlett et al. 2002). CB_1_ is predominantly expressed in the central nervous system (CNS), while CB_2_ is primarily expressed in the periphery, especially in immune cells (Pertwee 2007).

Endogenous ligands (“endocannabinoids”) produced in mammalian tissues target these receptors, and together with the catabolic and metabolic enzymes and transporter systems they constitute the endocannabinoid system.

Multiple drug therapy is often used with a single patient. As THC/CBD spray is indicated for MS and potentially advanced cancer pain, the likelihood is high that patients would be receiving different concomitant medications. As such, drug-drug interactions could occur which affect the bioavailability of THC/CBD spray through absorption, metabolism or disposition. In turn this could affect the treatment and adverse events (AEs) experienced by the patient (Chen & Raymond 2006). In some incidences, AEs experienced due to drug-drug interactions can be life-threatening, therefore understanding the mechanisms of these interactions is important so that dosing and safety information can be adjusted accordingly.

Cytochrome P450 (CYP450) is a family of isoenzymes responsible for the biotransformation of several drugs, and drug metabolism via this system has emerged as an important determinant of the occurrence of several drug-drug interactions that can result in toxicity, reduced pharmacological effect and AEs (Guengerich 2008). Determining whether the drugs involved act as enzyme substrates, inducers, or inhibitors can prevent clinically significant interactions from occurring. Moreover, avoiding co-administration or adjusting a patient's drug regimen early in the course of therapy can provide optimal response with minimal AEs (Ogu & Maxa 2000). Many different CYP450 isoenzymes have been identified to-date, including six which play important roles in drug metabolism (DiPiro 1999; Cupp & Tracy 1998): CYP1A2, CYP2C19, CYP2C9, CYP2D6, CYP2E1, and CYP3A4.

CYP450 inhibitors and inducers are known to affect the metabolism of THC. Previous literature reports have indicated that CBs, especially THC, are metabolized by CYP3A4, 2C9, 2C19 and possibly 2D6 in humans (Huestis 2007), and that the primary metabolites of THC and CBD are 11-hydroxy-THC (11-OH-THC) and 7-hydroxy-CBD, respectively (Huestis 2007). The formation of 11-OH-THC has been reported to be primarily catalysed by CYP2C19 and 2C9 (Bland et al. 2005).

In vitro studies of THC and CBD on CYP450 induction and inhibition indicate that both inhibit CYP1A1, 1A2 and 1B1 enzymes (Yamaori et al. 2010). CBD also has an inhibitory effect on CYP3A4 and CYP2C19. However, this effect only occurred at high concentrations (IC_50_ = 6-9 μM) of CBD (GW unpublished data), and in normal dosing, peak plasma concentrations of CBD are approximately 5 ng/mL or less, 400-fold lower than the levels at which CYP inhibition may be anticipated. As such, it is unlikely that THC/CBD spray would cause a relevant inhibition of CYP450s. However, to investigate the potential interactions of THC and CBD with drugs which also interact with the CYP450s CYP3A4 and CYP2C19, various known inducers/inhibitors of these isoenzymes were employed and the pharmacokinetics (PKs) of their co-administration with THC/CBD spray evaluated.

Rifampicin is an antibiotic drug, a strong inducer of CYP3A4 and moderate inducer of CYPs 2C19, 2B6, 2C8 and 2C9, and has been extensively used in clinical studies as a prototypical inducer of these enzymes (Division of Clinical Pharmacology 2012; Federal Drug Association 2012). Ketoconazole is a synthetic antifungal drug, is a strong inhibitor of CYP3A4 (Federal Drug Association 2012), and a weak inhibitor of CYPs 2C8 and 2C19 (Federal Drug Association 2012). Omeprazole is a proton-pump inhibitor which is primarily metabolized by, and demonstrates high affinity for CYP2C19 (Furuta et al. 2005), and is also a moderate inhibitor of 2C19 (Federal Drug Association 2012). This study investigated the potential interaction of these CYP450 inhibitors/inducers on the PK and safety profile of THC/CBD spray in healthy male subjects.

## Methods

This open-label, randomized, crossover, drug-interaction study took place at one study site in the UK (Quintiles Drug Research Unit at Guys Hospital), was approved by Guy's Research Ethics Committee, and was conducted according to the International Conference on Harmonisation guidelines on Good Clinical Practice and the ethical principles stated in the Declaration of Helsinki and local UK regulations. All participants gave written informed consent.

### Study design and treatment groups

A total of 36 healthy males subjects enrolled and were divided into three groups of 12. Within each group participants were randomized to one of two treatment sequences with six subjects receiving each sequence. Subjects received four sprays of THC/CBD spray (10.8/10 mg) alongside single usual daily doses of either rifampicin (600 mg), ketoconazole (400 mg) or omeprazole (40 mg) according to the following sequences, designed with a time-frame that was standard and fitting to the aims of this study:

- Sequence 1A. Subjects received a single dose of 4 sprays THC/CBD on Day 1 and once daily rifampicin on Days 2-10. Subjects then received both THC/CBD spray and rifampicin on Day 11.
- Sequence 1B. Subjects received rifampicin on Days 1-9. Subjects received THC/CBD spray and rifampicin on Day 10 and then a single dose of 4 sprays THC/CBD on Day 18.
- Sequence 2C. Subjects received a single dose of 4 sprays THC/CBD on Day 1 and once daily ketoconazole on Days 2-5. Subjects then received both THC/CBD spray and ketoconazole on Day 6.
- Sequence 2D. Subjects received ketoconazole on Days 1-4. Subjects received THC/CBD spray and once daily ketoconazole on Day 5. Subjects then received a single dose of 4 sprays THC/CBD on Day 10.
- Sequence 3E. Subjects received a single dose of 4 sprays THC/CBD on Day 1. Subjects received once daily omeprazole on Days 2-6, and then THC/CBD spray and omeprazole on Day 7.
- Sequence 3F. Subjects received a once daily dose of omeprazole on Days 1-5. Subjects received both THC/CBD spray and omeprazole on Day 6. Subjects then received a single dose of 4 sprays THC/CBD on Day 9.

### Blood sampling procedure and plasma preparation

Blood samples were collected at specified times and stored on ice (except rifampicin PK samples which were stored in iced water) prior to processing and storage. Plasma samples were separated by centrifugation (approximately 2500 rpm × 15 minutes at 4°C). Samples were stored in 4 mL amber glass screw top glass vials with PTFE lined screw caps labelled with Guys Drug Research (Quintiles Limited) labels. The aliquots were stored in clearly labelled containers in a freezer set at or below -20°C, until shipped for assay. Samples were shipped on dry ice at the appropriate time-points.

### Analysis method

The assay validation was undertaken by Advanced Bioanalytical Service Laboratories (London, UK), who developed the technique after reviewing the literature, which was based on the methodology adopted by three different groups (Foltz et al. 1983; Goodall & Basteyns 1995; Kemp et al. 1995), and designed with reference to FDA guidelines for industry (FDA Guidance for Industry 2012). The method utilised protein precipitation, solvent extraction and derivatisation for the sample preparation and then sample analysis by capillary gas chromatography and detection by a mass spectrometer (GC-MS). The validation procedure investigated the calibration model with the best regression fit over the concentration range 0.1 - 100 ng/mL for CBD, THC and 11-OH-THC, as well as precision and accuracy of the method, stability, carry-over, and specificity.

Human plasma from healthy volunteers was used to prepare the standards and quality control (QC) samples, with analytes extracted using hexane/ethyl acetate (7:1 ratio), derivatised with N,O-Bis(trimethylsilyl)trifluoroacetamide. Analytic grade THC (Sigma, UK), CBD (Sigma, UK) and 11-OH-THC (Radian International and Cerillant, UK) were obtained, and three sets of CBD, THC and 11-OH-THC were used to support the study. Deuterated THC-d_3_ (Sigma, UK) was used as the internal standard. The GC-MS equipment was a Hewlett Packard 6890 Gas Chromatograph attached to a Hewlett Packard 5973 Mass Selective Detector. Data handling was carried out using an MS Chemstation System and the peak area ratio of the analytes to the internal standard was calculated in Excel (2000). The concentrations were calculated from the ratio data using least squares ln(y) on ln(x) regression performed in Excel (2000), and were then checked manually. Regression analysis was undertaken to find the regression model that best described the calibration data (for details, see (Miller & Miller 1992)).

Intra-assay precision and bias was examined using spiked control samples analysed in replicates of five. Inter-assay precision and accuracy were analysed in quintuplet at three concentrations and on three separate occasions.

The lowest and upper Limits of Quantification (LOQ) were investigated by looking at five (500 μL) plasma samples containing 0.10 ng/mL and 100.0 ng/mL (the lowest and highest calibrators) of THC, CBD and 11-OH-THC, assayed in one batch, possessing acceptable precision and accuracy. As such, these lowest and upper LOQ were deemed suitable for the measurement of these analytes in human plasma over these concentration ranges.

The inter- and intra-assay accuracy of the assay calculated for THC was -0.53, -0.45, -1.72% and -0.50, -2.10, -0.86% at plasma THC concentrations of 2.0, 20.0 and 80.0 ng/mL, respectively. The inter- and intra-assay precision of the assay calculated for THC was 1.88, 2.51, 2.41% and 2.64, 1.09, 1.29% at plasma THC concentrations of 2.0, 20.0 and 80.0 ng/mL, respectively.

The inter- and intra-assay accuracy of the assay calculated for CBD was -2.90, 0.81, 1.78% and -2.00, -0.75, 2.38% at plasma CBD concentrations of 2.0, 20.0 and 80.0 ng/mL, respectively. The inter- and intra-assay precision of the assay calculated for CBD was 4.05, 2.28, 2.31% and 6.67, 1.43, 1.08% at plasma CBD concentrations of 2.0, 20.0 and 80.0 ng/mL, respectively.

The inter- and intra-assay accuracy of the assay calculated for 11-OH-THC was 0.00, 0.20, -3.44% and -0.46, -0.05, -1.43% at plasma 11-OH-THC concentrations of 2.17, 21.70 and 86.80 ng/mL, respectively. The inter- and intra-assay precision of the assay calculated for 11-OH-THC was 3.45, 3.44, 2.57% and 6.04, 1.96, 1.24% at plasma 11-OH-THC concentrations of 2.17, 21.70 and 86.80 ng/mL, respectively.

### Extraction procedure

A 0.5 mL aliquot of test sample, QC or blank plasma was placed into a test tube. The blank plasma was spiked with 50 μL of the appropriate standard solution to produce the calibration standards. 50 μL of the internal standard was added to each test tube. The samples were then diluted by the addition of 500 μL of 0.1% (w/v) ascorbic acid and the protein precipitated by the addition of 1.0 mL of acetonitrile. The proteins were removed by centrifugation and the supernatant concentrated to 1 mL using nitrogen at 50ºC. The samples were then basified by the addition of 300 μL of 5M sodium hydroxide and the analytes extracted by the addition of 2.0 mL of 7:1 hexane:ethyl acetate. After mixing for 16 minutes the tubes were centrifuged and the top layer transferred into clean 3 dram vials. The solvent was removed using nitrogen at 70ºC and the dried extract resuspended in 100 μL of BSTFA and transferred to microvials, capped and placed at 70ºC for 30 minutes to derivatise the analytes. The microvials were then cooled and loaded onto the autosampler tray for analysis where 1 μL was injected onto the GC-MS system.

### Inclusion and exclusion criteria

#### Inclusion criteria

Eligible subjects were healthy males between 18 and 45 years of age with a Body Mass Index (BMI) of between 18 and 30 Kg/m^2^. Subjects had no clinically significant abnormal findings upon physical examination, 12-lead electrocardiogram (ECG), medical history, clinical laboratory at screening, or renal and hepatic function. Subjects were non-users of tobacco products and were negative for Human Immunodeficiency Viruses I and II, Hepatitis B surface antigen, and antibodies to the Hepatitis C virus. Eligible subjects had a negative urine screen for alcohol, drugs of abuse (screening only) and cotinine, and were using an appropriate barrier method of contraception in addition to a second method of barrier contraception being used by their partner for the study duration and for three months following administration of THC/CBD spray.

#### Exclusion criteria

Subjects with a history of significant cardiovascular, pulmonary, hepatic, renal, haematologic, gastrointestinal, endocrine, immunologic, dermatologic, neurologic, or psychiatric disorder were excluded. Those with a history of alcohol or drug abuse within two years of the study were also excluded; however, those with a history of previous cannabis use were not excluded if willing to abstain for the study duration, unless they had used cannabis or CB-based medicine within 30 days prior to receiving study medication. Subjects with an abnormal diet, who had made substantial changes to eating habits in the 30 day period prior to the study, or who had participated in another clinical trial in the 90 day period prior to study entry were also excluded. Subjects who used any prescription or over the counter medication within 14 and seven days of study onset, or during the study, respectively, were also excluded, as were subjects who had treatment with any known enzyme-altering agents within 30 days prior to or during the study. In addition, subjects who had a postural drop of 20 mmHg or more in systolic blood pressure at screening were excluded, as were subjects who had donated blood or plasma within 90 days of study initiation. Subjects with a known history of hypersensitivity or idiosyncratic reaction to the study drug or related compounds were also excluded.

#### Concomitant medication

If concomitant medication was taken during the study, a joint decision was be made by the investigator and sponsor if the subject should continue in the study. No subject was permitted to take medication during the time of sample collection.

### Dietary restrictions

Xanthines and alcohol were prohibited 48 hours prior to dosing days and throughout each period of sample collection. Grapefruit was prohibited 10 days prior to initial dosing and throughout the study.

### Study endpoints

#### Pharmacokinetic endpoints

The PK endpoints were mean peak plasma concentration (C_max_), area under the plasma concentration versus time curve (AUC), from time 0 to the last measurable concentration (AUC_(0-t)_), AUC to infinite time (AUC_(0-inf)_), time to peak plasma concentration (T_max_), half-time (t_1/2_), elimination rate constant (Kel), oral clearance (CL/F) and apparent volume of distribution following oral administration (Varea/F; THC and CBD only) of THC, 11-OH-THC and CBD following administration of THC/CBD spray alone or THC/CBD spray concomitantly with rifampicin, ketoconazole, and omeprazole.

#### Safety endpoints

The safety endpoints were blood pressure, heart rate, ECG, clinical laboratory data (haematology and biochemistry), urinalysis, AEs and concomitant medications, recorded at each visit.

### Statistical methods

#### Sample size

A total of 36 subjects were planned and analyzed, with 12 subjects in each treatment group. However, there was no formal sample size power calculation for this study.

#### PK parameters

Summary statistics of PK parameters and concentrations included all treated subjects. Only subjects completing the study (i.e. PK data available for THC/CBD spray and THC/CBD spray plus interacting drug), were included in the statistical analyses of the interaction effects of rifampicin (Group 1), ketoconazole (Group 2), and omeprazole (Group 3). Data from the 3 groups were analyzed separately and no comparisons were made between groups. For each group, the PK parameters C_max_, AUC_(0-t)_ and AUC_(0-inf)_ were statistically analyzed using an analysis of variance model (ANOVA, SAS PROC MIXED). The traditional two-period crossover design was implemented. The model included effects of treatment, period, sequence, and subject within sequence. The log-transformed AUC and C_max_ data was analyzed using a general linear mixed model. The model included fixed terms for treatment, sequence, period and a random term for subject within sequence. Point estimates and 90% confidence intervals (CIs) for the ratios of the treatment means were calculated. The two one-sided hypotheses were tested at a 5% level for C_max_, AUC_(0-t)_ and AUC_(0-inf)_ by constructing 90% CIs for the ratio of the treatment means. The 90% CIs were obtained from the antilogarithms of the lower and upper bounds of the 90% CIs for the differences in the least-squares means of the log-transformed data. No significant interaction with respect to the log-transformed C_max_, AUC_(0-t)_ and AUC_(0-inf)_ was concluded if the 90% CI of the ratio of the geometric means fell within the range of 0.80 to 1.25. The summaries and descriptive statistics were calculated using WinNonlin ^®^ Professional, version 4.1b and SAS ^®^, version 9.1.

## Results

Mean participant ages for sequences 1A, 1B, 2C, 2D, 3E, and 3F were 28.8, 25.8, 32.5, 23.7, 26.5 and 27.0 years of age, respectively. Mean BMIs for the same respective sequences were 26.2, 24.2, 26.8, 25.2, 23.3 and 24.3 Kg/m^2^, giving a similar demographic profile across treatment groups.

### Plasma concentrations and exposure

**Figure 1:**
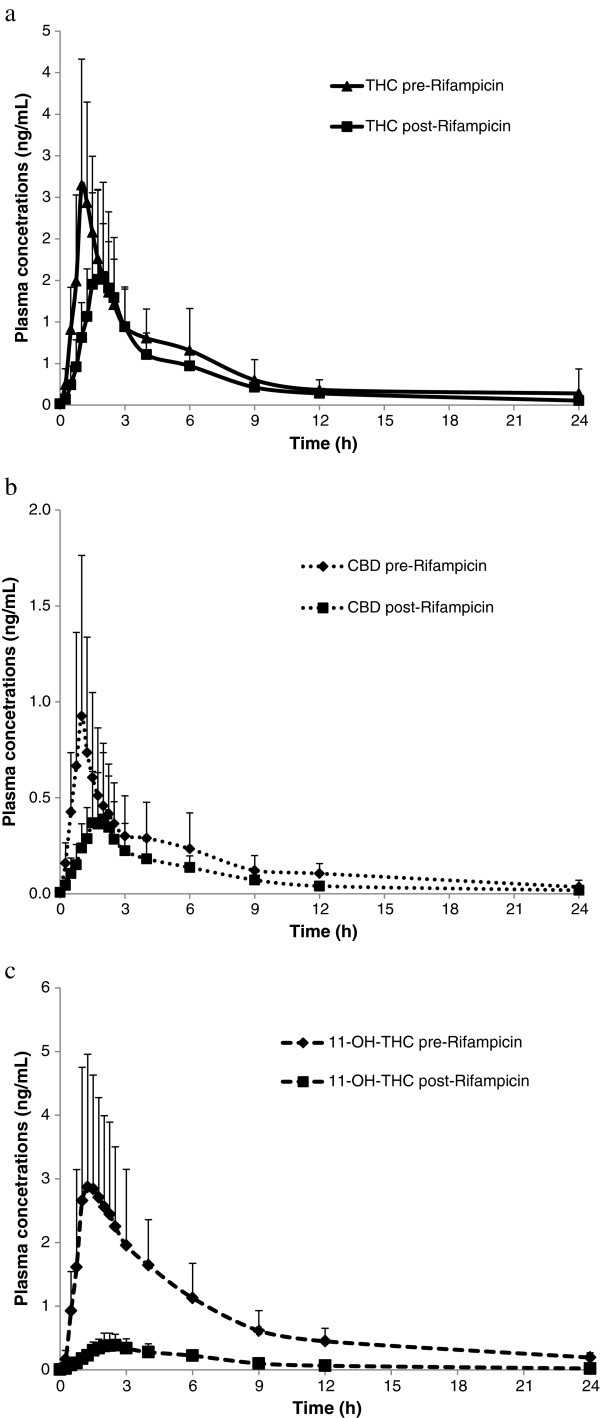
**Mean(+SD)plasma concentrations of THC(a),CBD(b)and 11**-**OH**-**THC(c)over time after administration of a single dose(4 sprays)of THC/CBD(*n =*11)or THC/CBD spray in combination with multiple dose(2 x 300 mg)administration of Rifampicin(*n*** = **12).**

**Figure 2:**
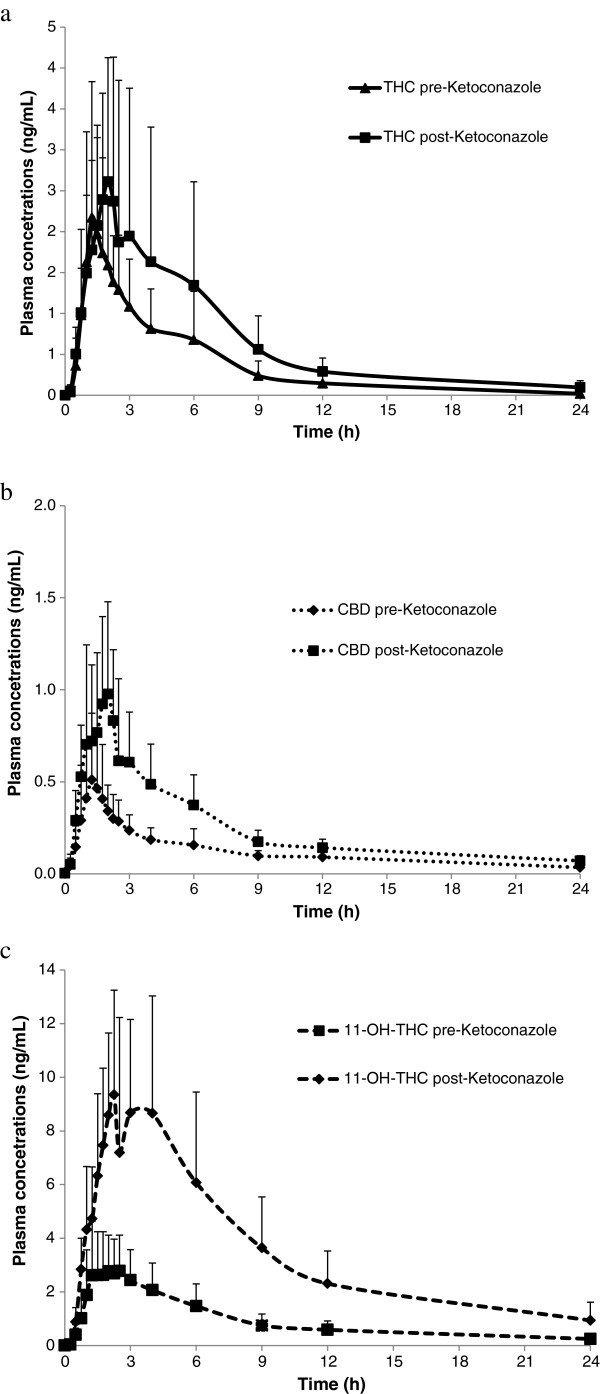
**Mean(+SD)plasma concentrations of THC(a),CBD(b)and 11-OH-THC(c)over time after administration of a single dose(4 sprays)of THC/CBD(*n =*11)or THC/CBD spray in combination with multiple dose(2 x 200 mg)administration of Ketoconazole (*n*= 11).**

**Figure 3:**
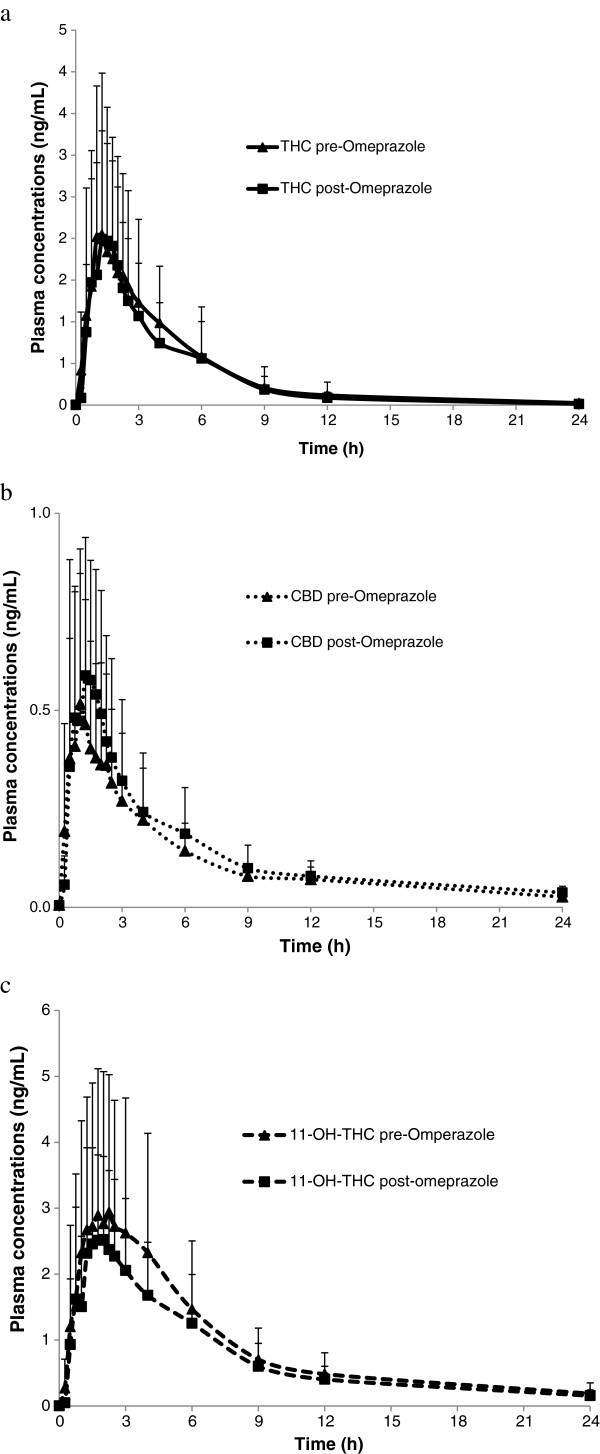
**Mean(+SD)plasma concentrations of THC(a),CBD(b)and 11-OH-THC(c)over time after administration of a single dose(4 sprays)of THC/CBD(*n =*11)or THC/CBD spray in combination with multiple dose(2 x 20 mg)administration of Omeprazole (*n*= 12).**

Mean plasma concentration versus time curves for THC, CBD and 11-OH-THC following administration of THC/CBD sprays alone and in combination with rifampicin, ketoconazole or omeprazole, are presented in Figures 1, 2 and 3, respectively.

Based on group mean C_max_ and AUC, the plasma exposure of THC, CBD and 11-OH-THC decreased for all three analytes following a single dose (4 sprays) of THC/CBD administered at the end of a 10-day dosing period (repeated dosing over 10 days) with rifampicin, compared with the PK parameters following a single dose of THC/CBD alone (4 sprays, no rifampicin). The decrease was general, occurring in 82-100% of subjects (Table 1).

**Table 1:** **Summary of pharmacokinetic parameters of THC**, **CBD and 11**-**OH**-**THC after a single dose of THC**/**CBD** (**4 sprays**) **alone or in combination with rifampicin**

Parameter	THC/CBD spray alone (***n =*** 12)	THC/CBD spray and rifampicin (***n =*** 12)
	**THC**	
AUC_(0-t)_ (h*ng/mL)	9.10 (2.98)	6.53 (2.70)
AUC_(0-inf)_ (h*ng/mL)	9.86 (3.35)^a^	7.53 (2.99)
C_max_ (ng/mL)	2.94 (1.21)	1.88 (1.07)
T_max_ (h)	1.01 (0.50-6.02)	1.75 (1.25-2.57)
Kel (h^-1^)	0.197 (0.093)^a^	0.189 (0.071)
t_½_ (h)	4.68 (3.42) ^a^	4.93 (3.91)
CL/F (L/h)	1207 (373)^a^	1595 (473)
Varea/F (L)	7625 (4326)^a^	10297 (5888)
	**CBD**	
AUC_(0-t)_ (h*ng/mL)	3.23 (2.13)	1.31 (0.89)
AUC_(0-inf)_ (h*ng/mL)	5.10 (3.06)	2.15 (0.94)^b^
C_max_ (ng/mL)	1.03 (0.81)	0.50 (0.37)
T_max_ (h)	1.00 (0.50-4.00)	1.50 (1.00-6.00)
Kel (h^-1^)	0.148 (0.108)	0.196 (0.092)^b^
t_½_ (h)	10.86 (12.71)	4.13 (1.65)^b^
CL/F (L/h)	2817 (1913)	5966 (3713)^b^
Varea/F (L)	28312 (19355)	34790 (20036)^b^
	**11**-**OH**-**THC**	
AUC_(0-t)_ (h*ng/mL)	18.61 (7.81)	1.84 (0.79)
AUC_(0-inf)_ (h*ng/mL)	21.59 (8.76)	2.78 (0.68)^c^
C_max_ (ng/mL)	3.38 (1.95)	0.45 (0.18)
T_max_ (h)	1.38 (0.50-6.02)	1.75 (1.50-2.57)
Kel (h^-1^)	0.083 (0.042)	0.189 (0.074)^c^
t_½_ (h)	9.88 (3.89)	4.19 (1.62)^c^

Data presented are mean and (SD) except for T_max_ where median and range are shown, ^a^n=11; ^b^n=10; ^c^n=11.

For the ketoconazole treatment group, mean C_max_ and AUC increased for all three analytes following a single dose of THC/CBD (4 sprays) administered at the end of a 5-day dosing period with ketoconazole compared with the PK parameters following a single dose of THC/CBD alone (4 sprays, no ketoconazole). The increase was also general, occurring in 63-100% of the subjects (Table 2).

**Table 2:** **Summary of pharmacokinetic parameters of THC**, **CBD and 11**-**OH**-**THC after a single dose of THC**/**CBD** (**4 sprays**) **alone or in combination with ketoconazole**

Parameter	THC/CBD spray alone (***n =*** 12)	THC/CBD spray and ketoconazole (***n =*** 11)
	**THC**	
AUC_(0-t)_ (h*ng/mL)	8.19 (5.67)	15.38 (13.43)
AUC_(0-inf)_ (h*ng/mL)	9.22 (5.94)^a^	16.76 (13.80)
C_max_ (ng/mL)	2.65 (1.32)	3.36 (1.65)
T_max_ (h)	1.50 (0.75-6.00)	1.75 (1.00-3.00)
Kel (h^-1^)	0.258 (0.092)^a^	0.189 (0.078)
t_½_ (h)	3.07 (1.31)^a^	4.43 (2.19)
CL/F (L/h)	1504 (688)^a^	920 (450)
Varea/F (L)	6328 (4164)^a^	5111 (2221)
	**CBD**	
AUC_(0-t)_ (h*ng/mL)	1.82 (1.03)	4.83 (2.01)
AUC_(0-inf)_ (h*ng/mL)	3.54 (0.80)^b^	6.50 (2.23)^c^
C_max_ (ng/mL)	0.66 (0.37)	1.25 (0.51)
T_max_ (h)	1.38 (0.75-6.00)	1.75 (1.00-2.52)
Kel (h^-1^)	0.122 (0.111)^b^	0.143 (0.066)^c^
t_½_ (h)	7.81 (3.00)^b^	6.54 (4.59)^c^
CL/F (L/h)	2998 (896)^b^	1731 (650)^c^
Varea/F (L)	31994 (12794)^b^	14349 (7076)^c^
	**11**-**OH**-**THC**	
AUC_(0-t)_ (h*ng/mL)	21.78 (11.34)	84.34 (40.18)
AUC_(0-inf)_ (h*ng/mL)	27.13 (13.34)^d^	95.26 (48.93)
C_max_ (ng/mL)	3.59 (1.67)	10.92 (3.83)
T_max_ (h)	1.50 (1.00-6.00)	2.25 (1.50-4.13)
Kel (h^-1^)	0.076 (0.014)^d^	0.095 (0.016)
t_½_ (h)	9.51 (2.18)^d^	7.48 (1.39)

Data presented are mean and (SD) except for T_max_ where median and range are shown, ^a^ n=11; ^b^n=9; ^c^n=10; ^d^n=11.

Following single dose administration of THC/CBD spray (4 sprays) at the end of a 6-day dosing period with omeprazole, the plasma concentrations and PK parameters were similar for THC, marginally higher for CBD and marginally lower for 11-OH-THC compared with the PK parameters following a single dose of THC/CBD alone (4 sprays, no omeprazole) (Table 3).

**Table 3:** **Summary of pharmacokinetic parameters of THC**, **CBD and 11**-**OH**-**THC after a single dose of THC**/**CBD** (**4 sprays**) **alone or in combination omeprazole**

Parameter	THC/CBD spray alone (***n =*** 12)	THC/CBD spray and omeprazole (***n =*** 12)
	**THC**	
AUC_(0-t)_ (h*ng/mL)	8.76 (8.62)	7.41 (4.75)
AUC_(0-inf)_ (h*ng/mL)	9.39 (8.81)	8.10 (4.78)
C_max_ (ng/mL)	2.50 (1.85)	2.48 (1.06)
T_max_ (h)	1.25 (0.77-3.02)	1.25 (1.00-1.75)
Kel (h^-1^)	0.305 (0.109)	0.357 (0.218)
t_½_ (h)	2.65 (1.25)	2.37 (0.92)
CL/F (L/h)	2161 (1990)	2284 (2520)
Varea/F (L)	6889 (5296)	6052 (3384)
	**CBD**	
AUC_(0-t)_ (h*ng/mL)	1.83 (1.19)	2.25 (1.51)
AUC_(0-inf)_ (h*ng/mL)	3.00 (1.43)^a^	3.33 (1.77)
C_max_ (ng/mL)	0.63 (0.43)	0.73 (0.30)
T_max_ (h)	1.15 (0.50-3.02)	1.25 (0.48-1.75)
Kel (h^-1^)	0.224 (0.158)^a^	0.210 (0.114)
t_½_ (h)	5.22 (4.51)^a^	5.46 (6.13)
CL/F (L/h)	4741 (3845)^a^	4772 (4550)
Varea/F (L)	26298 (14532)^a^	24757 (16311)
	**11**-**OH**-**THC**	
AUC_(0-t)_ (h*ng/mL)	21.52 (14.76)	17.69 (9.05)
AUC_(0-inf)_ (h*ng/mL)	24.17 (16.47)	19.80 (9.74)
C_max_ (ng/mL)	3.48 (2.27)	2.84 (1.40)
T_max_ (h)	2.00 (1.25-3.02)	1.52 (1.23-2.50)
Kel (h^-1^)	0.114 (0.062)	0.095 (0.029)
t_½_ (h)	7.30 (2.87)	7.98 (2.82)

Data presented are mean and (SD) except for T_max_ where median and range are shown, ^a^n=11.

### Oral clearance

The mean CL/F of THC/CBD spray increased from 1207 L/h to 1595 L/h (+32%) for THC and from 2817 L/h to 5966 L/h (+112%) for CBD after multiple dose rifampicin treatment (Table 1). Conversely, the mean CL/F of THC/CBD spray decreased from 1504 L/h to 920 L/h (-39%) for THC and from 2998 L/h to 1731 L/h (-42%) for CBD when co-administered with ketoconazole (Table 2). No significant differences in CL/F for THC or CBD were seen when THC/CBD spray was co-administered with omeprazole compared to THC/CBD spray alone (Table 3).

### T_max_ and t½

A small increase in median T_max_ for all analytes was observed when THC/CBD spray was co-administered with rifampicin (respective % changes for THC, CBD and 11-OH-THC of +73%, +50% and +27%) (Table 1). There was no change in the mean t_1/2_ of THC, but a general decrease for CBD and 11-OH-THC was observed (respective % changes for THC, CBD and 11-OH-THC of +5%, -62% and -58%) (Table 1). When THC/CBD spray was co-administered with ketoconazole, there was a small increase in mean T_max_ for 11-OH-THC and CBD, but no trend was observed for THC when looking at individual data (respective % changes for THC, CBD and 11-OH-THC of +17%, +27% and +50%) (Table 2). There was no change in the t_1/2_ of THC or CBD, but a general decrease for 11-OH-THC from 9.51 (THC/CBD spray alone) to 7.48 h (THC/CBD spray and ketoconazole) was observed (respective % changes for THC, CBD and 11-OH-THC of +44%, -16% and -21%) (Table 2). When THC/CBD spray was co-administered with omeprazole, the t_1/2_ and T_max_ for all analytes were similar to respective THC/CBD spray alone values (respective % changes in t_1/2_ for THC, CBD and 11-OH-THC of -11%, +5% and +9%; respective % changes in T_max_ for THC, CBD and 11-OH-THC of 0%, +9% and -24%%) (Table 3).

### Elimination rate constant

Kel was estimated over a short sampling period (less than two t_1/2_s) for the majority of CBD and 11-OH-THC profiles, and the regression had low precision (rsq adjusted <0.7) in a few profiles. Furthermore, AUC_(0-inf)_ had an extrapolated area larger than 20% for several CBD profiles (Table 1).

### 90% confidence intervals

Following co-administration of THC/CBD spray with rifampicin or ketoconazole, the 90% CIs for the geometric mean ratios of C_max_, AUC_(0-t)_ and AUC_(0-inf)_ did not fall within the pre-defined “no interaction” range of 0.80 to 1.25 for any of the analytes (Table 4).

**Table 4:** **Point estimate and 90**% **confidence interval for the geometric mean ratios of C**_**max**_, **AUC**_(**0**-**t**)_**and AUC**_(**0**-**inf**)_**for THC**/**CBD spray in combination with rifampicin**, **ketoconazole and omeprazole**

			90% confidence limits	
Analyte/PK variable	Number of subjects	Estimate	Lower	Upper
	**Rifampicin and THC**/**CBD spray**			
THC/C_max_	12	0.614	0.525	0.719
THC/AUC_(0-t)_	12	0.711	0.617	0.820
THC/AUC_(0-inf)_	11	0.761	0.667	0.868
CBD/C_max_	12	0.480	0.420	0.550
CBD/AUC_(0-t)_	12	0.381	0.274	0.529
CBD/AUC_(0-inf)_	10	0.422	0.265	0.673
11-OH-THC/C_max_	12	0.140	0.120	0.163
11-OH-THC/AUC_(0-t)_	12	0.099	0.088	0.113
11-OH-THC/AUC_(0-inf)_	11	0.131	0.115	0.150
	**Ketoconazole and THC**/**CBD spray**			
THC/C_max_	11	1.252	1.043	1.503
THC/AUC_(0-t)_	11	1.770	1.385	2.263
THC/AUC_(0-inf)_	10	1.840	1.446	2.342
CBD/C_max_	11	1.961	1.497	2.569
CBD/AUC_(0-t)_	11	2.715	2.047	3.601
CBD/AUC_(0-inf)_	9	1.923	1.560	2.370
11-OH-THC/C_max_	11	3.074	2.705	3.493
11-OH-THC/AUC_(0-t)_	11	3.823	3.405	4.292
11-OH-THC/AUC_(0-inf)_	10	3.616	3.181	4.111
	**Omeprazole and THC**/**CBD spray**			
THC/C_max_	12	1.125	0.842	1.501
THC/AUC_(0-t)_	12	0.964	0.643	1.445
THC/AUC_(0-inf)_	12	0.962	0.668	1.386
CBD/C_max_	12	1.320	0.938	1.859
CBD/AUC_(0-t)_	12	1.326	0.756	2.325
CBD/AUC_(0-inf)_	11	1.096	0.745	1.611
11-OH-THC/C_max_	12	0.866	0.700	1.073
11-OH-THC/AUC_(0-t)_	12	0.869	0.629	1.201
11-OH-THC/AUC_(0-inf)_	12	0.869	0.641	1.179

Following co-administration of THC/CBD spray with omeprazole, for THC and CBD the upper limit of the 90% CI of the geometric mean ratio of C_max_ was above 1.25. The AUC_(0-t)_ and AUC_(0-inf)_ 90% CI upper limits were above 1.25 and the lower limits were below 0.80 for THC and CBD (Table 4). For 11-OH-THC the lower limits of the 90% CI of C_max_, AUC_(0-t)_ and AUC_(0-inf)_ were below 0.80, did not fall within the pre-defined “no interaction” range of 0.80 to 1.25 (Table 4).

### Inter-subject variability

The C_max_ and AUC % Co-efficient of Variation (CV%) ranges were similar for all analytes in all treatment groups with the exception of a higher CV% for THC when THC/CBD spray was administered alone in the THC/CBD spray alone or in combination with omeprazole treatment group.

THC/CBD spray in combination with rifampicin had a CV% of 25-74% and for THC/CBD spray alone the CV% was 33-79% for all analytes. THC/CBD spray in combination with ketoconazole had a CV% of 34-87% and for THC/CBD spray alone this was 23-69% for all analytes. THC/CBD spray in combination with omeprazole had a CV% range of 40-67% for combination treatment and 48-69% for THC/CBD spray alone for CBD and 11-OH-THC. The CV% range for THC was higher at 74-98% for THC/CBD spray alone compared to 43-64% when administered in combination with omeprazole.

## Safety and tolerability

A summary of all treatment-emergent AEs (TEAEs) occurring in one or more subject is presented in Table 5. With the exception of the THC/CBD spray plus ketoconazole study group, all study medication was generally well tolerated by subjects.

**Table 5:** **Treatment emergent adverse events with an incidence** >**1**

Primary system organ class Preferred term*	No. of subjects (%)	No. of subjects (%)	No. of subjects (%)
	**THC**/**CBD spray** (***n*** = **36**)		
**Psychiatric disorders**			
Euphoric mood	2 (6)	-	-
**Nervous system disorders**			
Dizziness	2 (6)	-	-
Dizziness postural	3 (8)	-	-
Headache	5 (14)	-	-
Somnolence	11 (31)	-	-
**Gastrointestinal disorders**			
Nausea	2 (6)	-	-
	**Rifampicin(*n*** = **12)**	**THC**/**CBD spray and Rifampicin(*n*** = **12)**	**THC**/**CBD spray(*n*** = **12)**
**Nervous system disorders**			
Dysgeusia	1 (8)	0	1 (8)
Headache	0	1 (8)	1 (8)
Somnolence	0	0	2 (17)
	**Ketoconazole(*n*** = **12)**	**THC**/**CBD spray and Ketoconazole(*n*** = **11)**	**THC**/**CBD spray(*n*** = **12)**
**Nervous system disorders**			
Somnolence	0	4 (36)	4 (33)
Dizziness	0	3 (27)	2 (17)
Lethargy	0	3 (27)	0
Dysgeusia	0	2 (18)	0
Headache	0	2 (18)	0
Somnolence	0	1 (9)	1 (8)
**Psychiatric disorders**			
Euphoric mood	0	7 (64)	0
Anxiety	0	1 (9)	1 (8)
Disorientation	0	1 (9)	1 (8)
	**Omeprazole(*n*** = **12)**	**THC**/**CBD spray and Omeprazole(*n*** = **12)**	**THC**/**CBD spray(*n*** = **12)**
**Nervous system disorders**			
Dizziness	0	3 (25)	0
Dizziness postural	0	0	2 (17)
Headache	1 (8)	0	4 (33)
Somnolence	0	2 (17)	5 (42)
**Gastrointestinal disorders**			
Dry mouth	0	2 (17)	1 (8)

*MedDRA version 10.0.

The most common TEAEs with THC/CBD spray alone were somnolence and headache. For THC/CBD spray plus rifampicin, these were rhinitis, headache and malaise; but none was considered related to THC/CBD spray, all were of mild severity, and none was reported with a subject incidence greater than one. Subjects receiving THC/CBD spray plus ketoconazole reported the greatest subject incidence (100%) of TEAEs in the study, with a total of 35 TEAEs reported in 11 subjects. The majority of these were of the system organ class (SOC) nervous system disorders. Similarly, the majority of TEAEs reported by subjects receiving THC/CBD spray plus omeprazole were also nervous system disorders, the most common being dizziness.

No other clinically significant abnormalities for laboratory safety measurements or ECG parameters were reported during the study.

## Discussion

This study investigated the effects of the known CYP450 inhibitors or inducers rifampicin, ketoconazole and omeprazole, on the PK and safety profiles of THC/CBD spray.

### Rifampicin and THC/CBD spray

When prescribing drugs that share the CYP3A4 pathway, plasma levels should be periodically monitored, otherwise it is possible that drug levels may reach a toxic state that can manifest as serious medical events if one of them is a CYP3A4 inhibitor (such as ketoconazole) (Ogu & Maxa 2000). If one of the drugs is a CYP3A4 inducer, such as rifampicin, then the effectiveness of the other drug might be compromised following a more rapid reduction than usual of the plasma level. As such, investigating the PKs of THC/CBD spray in combination with a CYP3A4 inducer was of high clinical importance. Rifampicin was chosen as it has been extensively used in clinical studies as a prototypical inducer of CYP3A4 (Division of Clinical Pharmacology 2012), but it is also a moderate CYP2C19 inducer (Federal Drug Association 2012). Overall exposure to repeated daily doses of rifampicin for 10 days, followed by a single dose of THC/CBD spray (4 sprays), reduced the mean plasma levels of THC and CBD, and most of all, 11-OH-THC, as compared with levels observed when THC/CBD spray alone was administered. While the magnitude of the reduction in the plasma levels of the three analytes was within the range of inter-individual variation observed with THC/CBD spray alone, suggesting that significant induction of CYP3A4 would lead to only a slight reduction in exposure to CBs, intra-subject variability must also be considered. For example, should a subjects whose symptoms are controlled by a daily dose of seven sprays of THC/CBD spray experience a reduction in plasma levels of CBs of 1/3 in the presence of a CYP3A4 inducer, they would need to increase their dose to experience the same efficacy of THC/CBD spray. The effect of rifampicin on the group mean C_max_ and AUC of CBD and THC was consistent with the inducing effects of rifampicin on the CYP3A4 isoenzyme (Michalets 1998). Both CBD and THC exposure decreased when rifampicin was administered with THC/CBD spray and the apparent clearance for these analytes increased after multiple dose administration of rifampicin. Although an increase in exposure of the 11-OH-THC metabolite was expected due to anticipated conversion of THC to 11-OH-THC via CYP3A4, instead a significant decrease in the metabolite exposure was observed, suggesting that CYP3A4 is also involved in the further metabolism of the primary metabolite, 11-OH-THC. A suggested mechanism for this effect is that increased CYP3A4 activity induced by rifampicin induced further metabolism of 11-OH-THC, causing a reduced plasma level of 11-OH-THC. This is the first investigation in humans to identify CYP3A4 as a significant mediator of 11-OH-THC metabolism, an interesting finding for this psychoactive THC metabolite.

#### Ketoconazole and THC/CBD spray

Similarly, the effects of the potent CYP3A4 inhibitor ketoconazole on the PKs of THC/CBD spray were also investigated. For both THC and CBD, there was an increase in mean C_max_ and AUC following THC/CBD spray administration after 5-day period of daily administration of ketoconazole. These increases in mean values were considerably less than the range of inter-subject variability for the same parameters. However, intra-subject variability must also be considered, in that should an individual experienced enhanced plasma levels of CBs in the presence of a CYP3A4 inhibitor, then their daily dose of THC/CBD spray would need to be reduced accordingly in order to balance the efficacy of the compound against any adverse effects that may occur at higher doses.

For 11-OH-THC, the C_max_ was 3.1-times higher after CYP3A4 inhibition by ketoconazole, and the AUC was 3.8-times higher, confirming that 11-OH-THC is likely to be metabolized predominantly by CYP3A4. The effect of ketoconazole on the group mean C_max_ and AUC of CBD and THC was consistent with the inhibitory effects of ketoconazole on the CYP3A4 isoenzyme. Both CBD and THC exposure increased when THC/CBD spray was administered with ketoconazole and the apparent clearance for CBD and THC decreased after multiple dose administration of ketoconazole. Although a decrease in exposure of the 11-OH-THC metabolite was expected due to inhibition of the conversion of THC to 11-OH-THC via CYP3A4, a significant increase in metabolite exposure was observed which again suggests CYP3A4 is involved in the metabolism of the metabolite, 11-OH-THC.

#### Omeprazole and THC/CBD spray

To investigate the potential interaction of THC/CBD spray with a CYP2C19 inhibitor, omeprazole was employed. In contrast to rifampicin and ketoconazole, co-administration of THC/CBD spray with omeprazole had no apparent affect the group mean C_max_ or AUC results for any of the analytes. Any slight differences group mean AUC and C_max_ values for all three analytes were non-significant and well within the range of inter-individual variation observed with THC/CBD spray alone. Furthermore, the apparent clearance for THC and CBD was not changed between THC/CBD spray alone versus THC/CBD spray and omeprazole treatments. However, a decrease in exposure to 11-OH-THC was observed following co-administration of omeprazole with THC/CBD spray. Nevertheless, examining the individual exposure data, no clear conclusion could be drawn with respect to any effect of omeprazole. These findings suggest that THC, CBD are not substrates for the CYP2C19 isoenzyme.

#### Safety and tolerability

THC/CBD spray was generally well tolerated when given alone, illustrated by the fact that there were no serious AEs during the study. The majority of TEAEs were mild in severity and, as expected, of the SOC of nervous system disorders, with somnolence being the most commonly reported. While the exposure to THC/CBD spray was lower than usual in chronic patients, the administration procedure did not follow the slow up-titration process that takes 4-5 days to reach a dose of 4 sprays/day (and goes on for 7-8 days to reach the average 6-7 sprays/day dose, and up to 14 days to reach the maximum dose of 12 sprays/day). As such, the incidence of AEs in the SOC of nervous disorders in the current study is not surprising. A review of collated results from initial randomized-controlled clinical trials with THC/CBD spray involving 930 patients demonstrated that the most frequently reported AEs (incidence >10%) were dizziness (28%), diarrhoea (13%), fatigue (11%) and nausea (11%) (Constantinescu & Sarantis 2006). Recent publications also demonstrate that AEs the SOC of nervous systems disorders were among the commonly occurring AEs reported with THC/CBD spray use (Johnson et al. 2012; Langford et al. 2013).

No difference was observed in the proportions of subjects reporting AEs upon co-administration of THC/CBD spray with rifampicin or omeprazole compared with THC/CBD spray alone. Again, all AEs were of mild severity, with headache and dysguesia being the most commonly reported for THC/CBD spray and rifampicin (one subject), and dizziness in three subjects taking THC/CBD spray and omeprazole.

Although all subjects receiving THC/CBD spray and ketoconazole experienced AEs, this combination was still relatively well tolerated, and all but one AE was of mild severity. The majority of TEAEs were classed as nervous system disorders, including somnolence and dizziness which occurred at the same incidence as with THC/CBD spray alone. However, there was an increased incidence of euphoric mood, lethargy, dygeusia and headache when THC/CBD spray was given in combination with ketoconazole. Only one event was classed as moderate in terms of severity (anxiety) and the event resolved without intervention. All other events were of mild severity. Notably in this group, all subjects had increases in C_max_ for 11-OH-THC, and seven had an increase in THC C_max_, with nine subjects also having an increase in AUC. Taking into account the increase in exposure after combined administration compared to THC/CBD spray alone, it is possible that the difference in PKs may account for the increase in CNS-type AEs observed.

#### Study limitations

There were a number of limitations to this study. Kel was estimated over a short sampling period (less than two t_1/2_s) for the majority of profiles in this study and the regression had low precision in some profiles. AUC_(0-inf)_ had an extrapolated area which was larger than 20% for some profiles. However, despite these limitations, the AUC_(0-inf)_ and the AUC_(0-t)_ were in agreement, and the statistical results are thus considered reliable. Further consistency and validity of the study is demonstrated by the similarity of the PK exposure parameters following a single dose of THC/CBD spray in the absence of any inducer or inhibitor, which are in good agreement with the PK data provided in a previous clinical study performed by the authors (ref PK study). The inter-subject variability was substantial and greater than the difference in exposure means before and after rifampicin, ketoconazole, or omeprazole treatment, suggesting that any effect of other medications metabolized by relevant CYP enzymes on THC/CBD spray is likely to be within the normal range of variation. However, there was generally a similar variability in the group mean primary PK parameters between the THC/CBD spray alone and the THC/CBD spray plus inducer or inhibitor treatments.

Doses of THC/CBD (4 sprays instead the average 6-8 sprays/day) and intake patterns (4 sprays in a row instead of evenly distributed through the day) were not equivalent to those in the approved label. However, during a phase IIb dose ranging study in cancer patients with pain, in the low dose THC/CBD spray (1-4 sprays) group, over 90% of patients titrated to a dose of 3 or 4 sprays, leading to the conclusion that a minimal effective dose was 3 sprays per day (Porteney et al. 2012). Additionally, efficacy was observed in the low dose group, reaching statistical significance for the continuous response analysis (pain 0-10 Numerical Rating Scale score) and for the mean change from baseline in score, demonstrating that 4 daily sprays is a clinically relevant dose (Porteney et al. 2012). Distributing the dose throughout the day in the current study would have given a very different PK profile, and would not have been a suitable approach for the current study. Additionally, during a previous Phase I pharmacokinetics study, doses of 2 daily sprays of THC/CBD spray gave low C_max_ values (Stott et al. 2012). As such, a dose of 4 daily sprays was chosen to give good plasma concentration over time curves.

## Conclusions

In conclusion, inhibition of CYP2C19 by omeprazole did not significantly alter the PK of THC/CBD spray suggesting that THC, CBD and 11-OH-THC are not substrates for this isoenzyme at the clinically relevant dose of THC/CBD spray investigated. The CYP3A4 inducer rifampicin caused a decrease in exposure to all three analytes, although not extreme and within the natural range of variation between subjects. Conversely, the CYP3A4 inhibitor ketoconazole caused increased exposure to all analytes, suggesting that THC, CBD and 11-OH-THC all are substrates for this isoenzyme. Moreover, the findings with rifampicin and ketoconazole suggest that CYP3A4 is involved in the metabolism of 11-OH-THC. On the basis of these findings, there is likely to be little impact on other drugs metabolized by CYP enzymes on the PK parameters of THC/CBD spray, but potential effects should be taken into consideration when co-administering THC/CBD spray with compounds which share the CYP3A4 pathway. THC/CBD spray was also well tolerated in healthy subjects both alone and in combination with rifampicin, ketoconazole and omeprazole.

### Ethical standards

The current study was approved by Guy's Research Ethics Committee, and was conducted in accordance with the International Conference on Harmonisation guidelines on Good Clinical Practice and the ethical principles stated in the Declaration of Helsinki and local UK regulations. All participants gave written informed consent.

^†^Sativex ^®^, a THC/CBD oromucosal spray, does not have an INN. Nabiximols is the US Adopted Name (USAN).
